# Inhibition of Antiviral Innate Immunity by *Avibirnavirus* VP3 via Blocking TBK1-TRAF3 Complex Formation and IRF3 Activation

**DOI:** 10.1128/mSystems.00016-21

**Published:** 2021-05-11

**Authors:** Tingjuan Deng, Boli Hu, Xingbo Wang, Lulu Lin, Jianwei Zhou, Yuting Xu, Yan Yan, Xiaojuan Zheng, Jiyong Zhou

**Affiliations:** a MOA Key Laboratory of Animal Virology, Zhejiang University Center for Veterinary Sciences, Hangzhou, People’s Republic of China; b State Key Laboratory for Diagnosis and Treatment of Infectious Diseases, First Affiliated Hospital, Zhejiang University, Hangzhou, China; Princeton University

**Keywords:** innate antiviral response, TNF receptor-associated factor 3, *Avibirnavirus* VP3, ubiquitination

## Abstract

The host innate immune system develops various strategies to antagonize virus infection, and the pathogen subverts or evades host innate immunity for self-replication. In the present study, we discovered that *Avibirnavirus* infectious bursal disease virus (IBDV) VP3 protein significantly inhibits MDA5-induced beta interferon (IFN-β) expression by blocking IRF3 activation. Binding domain mapping showed that the CC1 domain of VP3 and the residue lysine-155 of tumor necrosis factor receptor-associated factor 3 (TRAF3) are essential for the interaction. Furthermore, we found that the CC1 domain was required for VP3 to downregulate MDA5-mediated IFN-β production. A ubiquitination assay showed that lysine-155 of TRAF3 was the critical residue for K33-linked polyubiquitination, which contributes to the formation of a TRAF3-TBK1 complex. Subsequently, we revealed that VP3 blocked TRAF3-TBK1 complex formation through reducing K33-linked polyubiquitination of lysine-155 on TRAF3. Taken together, our data reveal that VP3 inhibits MDA5-dependent IRF3-mediated signaling via blocking TRAF3-TBK1 complex formation, which improves our understanding of the interplay between RNA virus infection and the innate host antiviral immune response.

**IMPORTANCE** Type I interferon plays a critical role in the host response against virus infection, including *Avibirnavirus.* However, many viruses have developed multiple strategies to antagonize the innate host antiviral immune response during coevolution with the host. In this study, we first identified that K33-linked polyubiquitination of lysine-155 of TRAF3 enhances the interaction with TBK1, which positively regulates the host IFN immune response. Meanwhile, we discovered that the interaction of the CC1 domain of the *Avibirnavirus* VP3 protein and the residue lysine-155 of TRAF3 reduced the K33-linked polyubiquitination of TRAF3 and blocked the formation of the TRAF3-TBK1 complex, which contributed to the downregulation of host IFN signaling, supporting viral replication.

## Abstract (precis)

Type I interferon plays a critical role in the host response against virus infection, including *Avibirnavirus.* However, many viruses have developed multiple strategies to antagonize the innate host antiviral immune response during coevolution with the host. In this study, we first identified that K33-linked polyubiquitination of lysine-155 of TRAF3 enhances the interaction with TBK1, which positively regulates the host IFN immune response.

## INTRODUCTION

The detection of invading virus by the innate immune system depends on pattern recognition receptor-mediated recognition of viral nucleic acids (1). Retinoic acid-induced gene I (RIG-I)-like receptors (RLRs) and melanoma differentiation gene 5 (MDA5) detect viral RNA (2). Upon recognition of viral RNA, the downstream key adaptor mitochondrial antiviral signaling (MAVS) protein is oligomerized and activated by being recruited to the RLR signalosome, which leads to the activation of TBK1/IKKε and IKKα/β/γ complexes through tumor necrosis factor (TNF) receptor-associated factors 3 and 5 (TRAF3 and -5), respectively (3, 4). Ultimately, interferon regulatory factor 3 (IRF3) and nuclear factor κB (NF-κB) are activated and induce beta interferon (IFN-β) expression (5–7).

IFN-β is one of the major cytokines rapidly triggered by many viruses in infected cells and plays a critical role in the innate antiviral response (8). To combat the antiviral effects of IFN-β, many viruses, including *Avibirnavirus*, have evolved various elaborate mechanisms to subvert host innate immunity (9–11). Infectious bursal disease virus (IBDV), the representative member of the genus *Avibirnavirus* in the family *Birnaviridae*, contains a double-stranded RNA (dsRNA) genome comprising segments A and B and encodes RNA-dependent RNA polymerase VP1, two structural proteins (VP2 and VP3), a viral protease (VP4), and a nonstructural protein (VP5) (12–14). Reports show that VP4 and VP3 participate in the modulation of IFN-β production upon virus infection. VP4 attenuates IFN-β expression via binding to the glucocorticoid-induced leucine zipper, and VP3 competes with MDA5 to bind the intracellular viral genomic dsRNA to inhibit IFN-β production (11, 15). In addition, studies demonstrate that host cell factor Staufen1 (STAU1) and protein kinase R (PKR) can be employed by genomic dsRNA and viral protein VP3, respectively, to block IFN-β induction (16, 17). However, whether viral protein VP3 can antagonize IFN-β production by targeting the components of the RLR pathway is unknown.

TRAF3, a member of the TRAF family, is a positive regulator of IRF3 activation and type I IFN induction (6). Like other TRAF members, TRAF3 is conserved in vertebrates and contains an N-terminal RING finger, two TRAF-type zinc fingers, and a C-terminal TRAF-MATH domain (18). Upon RNA virus infection, TRAF3 undergoes K63-linked ubiquitination (Ub) in mitochondria, and the posttranslational modification of TRAF3 is essential in the virus-triggered induction of type I interferon (19). K48-linked ubiquitination guides TRAF3 for proteasome degradation (20). However, to date, the roles of other types of polyubiquitination of TRAF3 in the IFN signaling pathway are not clearly elucidated.

In the present study, we first revealed that the *Avibirnavirus* VP3 protein effectively inhibits MDA5-mediated IFN-β production through its CC1 domain (residues 11 to 24). Further analysis demonstrated that VP3 directly targeted the residue lysine-155 of TRAF3 to decrease the K33-linked polyubiquitination of TRAF3 and blocked the formation of the TRAF3-TBK1 complex to repress IFN-β production. To our knowledge, this is the first report of the recognition that K33-linked polyubiquitination of lysine-155 of TRAF3 may positively regulate the host innate immune response, which can be hijacked by *Avibirnavirus* VP3 protein to evade the host antiviral immune response.

## RESULTS

### Viral protein VP3 is a negative regulator of MDA5-driven IFN-β production.

**FIG 1:**
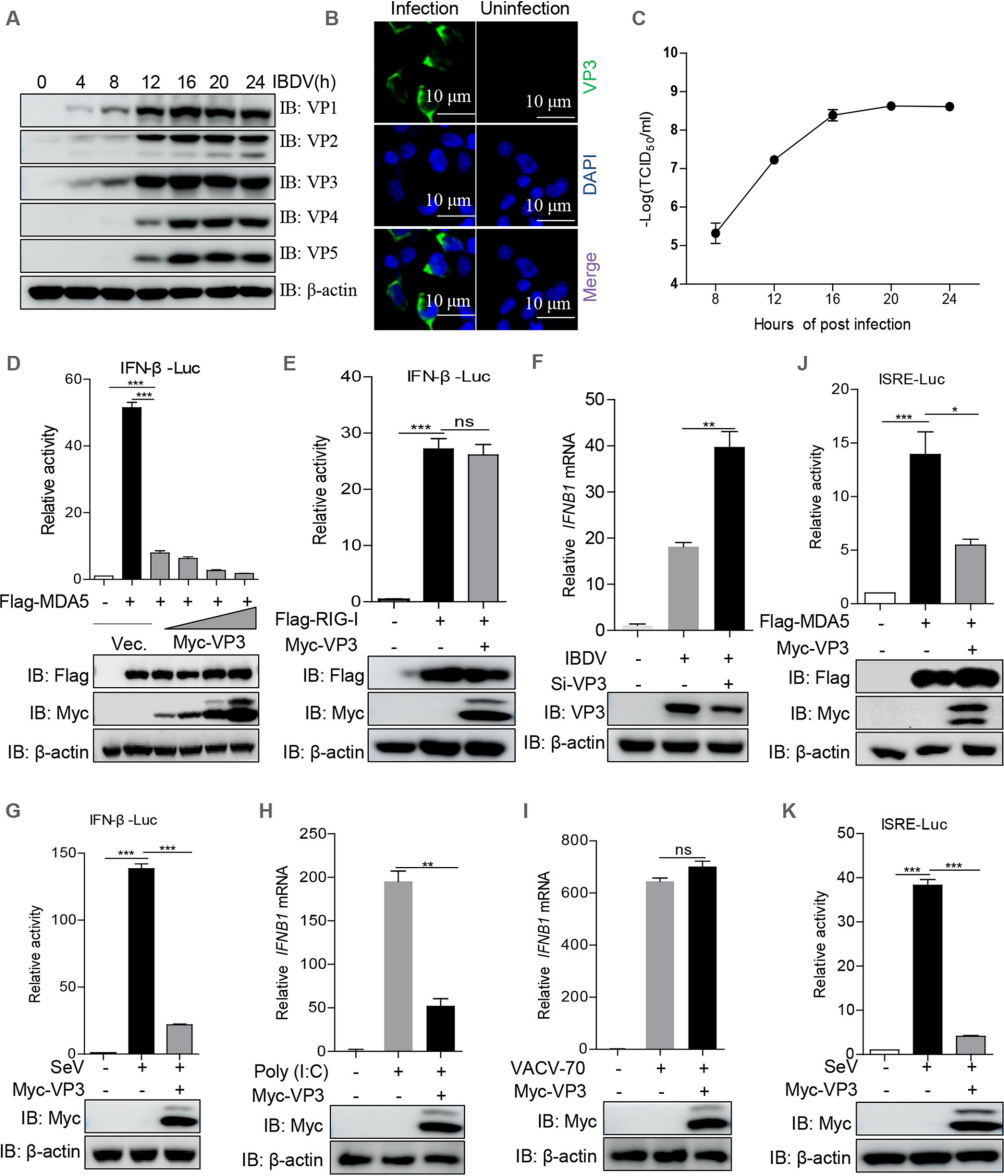
*Avibirnavirus* VP3 protein suppresses MDA5-mediated IFN-β activation. (A and B) Replication of IBDV strain NB in HEK293T cells. HEK293T cells were infected with IBDV NB at an MOI of 10 for the indicated times. (A) The expression levels of viral proteins were detected with the indicated viral protein antibodies. IB, immunoblotting. (B) At 12 h after IBDV infection, cells were subjected to an immunofluorescence staining assay with anti-VP3 mouse MAb. (C) Kinetics of IBDV NB replication in HEK293T cells. HEK293T cells were infected with IBDV at an MOI of 10. At different time points (8, 12, 16, 20, and 72 h) after IBDV infection, the viral titers in the cell cultures were harvested, and the TCID_50_ was calculated. (D and E) VP3 represses IFN-β promoter activity. Luc, luciferase; Vec., vector. (E and F) KEK293T cells were cotransfected with the IFN-β reporter and the pRL-TK and VP3 plasmids (100 ng, 200 ng, 300 ng, 500 ng) along with MDA5 (E) or RIG-I (F) for 36 h. Luciferase activity was then detected by a dual-luciferase assay. ns, not significant. (F) Viral protein VP3 knockdown contributes to the induction of IFN-β by IBDV. DF-1 cells were infected with IBDV at an MOI of 10. At 1 h after infection, the resulting cells were transfected with the RNAi sequence (GACGAAGUUGCCAAAGUCUAU) against VP3 for another 12 h. The mRNA of *IFNB1* was detected by qPCR. (G) VP3 overexpression inhibits SeV-induced IFN-β activation. The IFN-β reporter and the pRL-TK and VP3 plasmids were cotransfected into HEK293T cells. At 24 h after transfection, cells were mock infected or infected with SeV (20 hemagglutination units/well) for 10 h and then were subjected to a dual-luciferase assay. (H and I) Effects of VP3 on poly(I·C) (H)- and VACV-70 (I)-induced levels of *IFNB1* mRNA. The expression plasmid of VP3 or an empty vector was transfected into HEK293T cells. At 24 h after transfection, the cells were stimulated with poly(I·C) or VACV-70 for another 12 h. The *IFNB1* mRNA was measured by a quantitative PCR assay. (J and K) VP3 represses ISRE promoter activity induced by MDA5 (J) or SeV (K). Luciferase assays were performed as described for panels E and G. Data are presented as the means ± SD from three independent experiments. *, *P < *0.05; **, *P < *0.01; ***, *P < *0.001.

IBDV strains are adapted to replicate efficiently in multiple types of cells, including chicken B cells, CEF and DF-1 cells, and Vero and HEK293T cells (15, 21, 22). Here, we further confirmed that IBDV could efficiently replicate in HEK293T cells (Fig. 1A to C). IFN-β played a critical role in the host response against IBDV infection (23), and *Avibirnavirus* VP3 negatively modulated IFN-β production by competing with MDA5 to bind dsRNA (11). To explore whether viral protein VP3 inhibited IFN-β production by hijacking the components in the RLR pathway, we detected the effects of VP3 on RLR (MDA5 and RIG-I)-driven IFN-β promoter activation using a dual-luciferase reporter assay. Figure 1D and E showed that, in a comparison with an empty vector, VP3 overexpression significantly decreased MDA5-induced IFN-β promoter activity in a dose-dependent manner, but not that induced by RIG-I. Consistently, VP3 knockdown contributed to IBDV-induced IFN-β expression when the RNA interference (RNAi) sequence against *VP3* was transfected into DF-1 cells (Fig. 1F). Also, VP3 dramatically inhibited IFN-β promoter activation driven by Sendai virus (SeV), an RNA virus of the *Paramyxoviridae* family (Fig. 1G). Moreover, we observed that VP3 had a significant inhibitory effect on *IFNB1* transcription induced by poly(I·C), a long synthetic analog of dsRNA, but not that induced by VACV-70, a DNA ligand (Fig. 1H and I), suggesting that VP3 may only negatively modulate the MDA-5-driven IFN-β signaling pathway during RNA virus infection. Since the activation of IRF3 and NF-κB directly activate promoters of type I IFNs, such as IFN-β (24), we assessed the impact of VP3 overexpression on the IFN-stimulated response element (ISRE) luciferase reporter, which is sufficiently activated by IRF3 activation. As shown in Fig. 1J and K, VP3 markedly repressed ISRE reporter activity, indicating that VP3 may block IFN-β activation through the IRF3-mediated pathway.

### VP3 enhances viral replication by suppressing MDA5-induced IRF3 activation.

**FIG 2:**
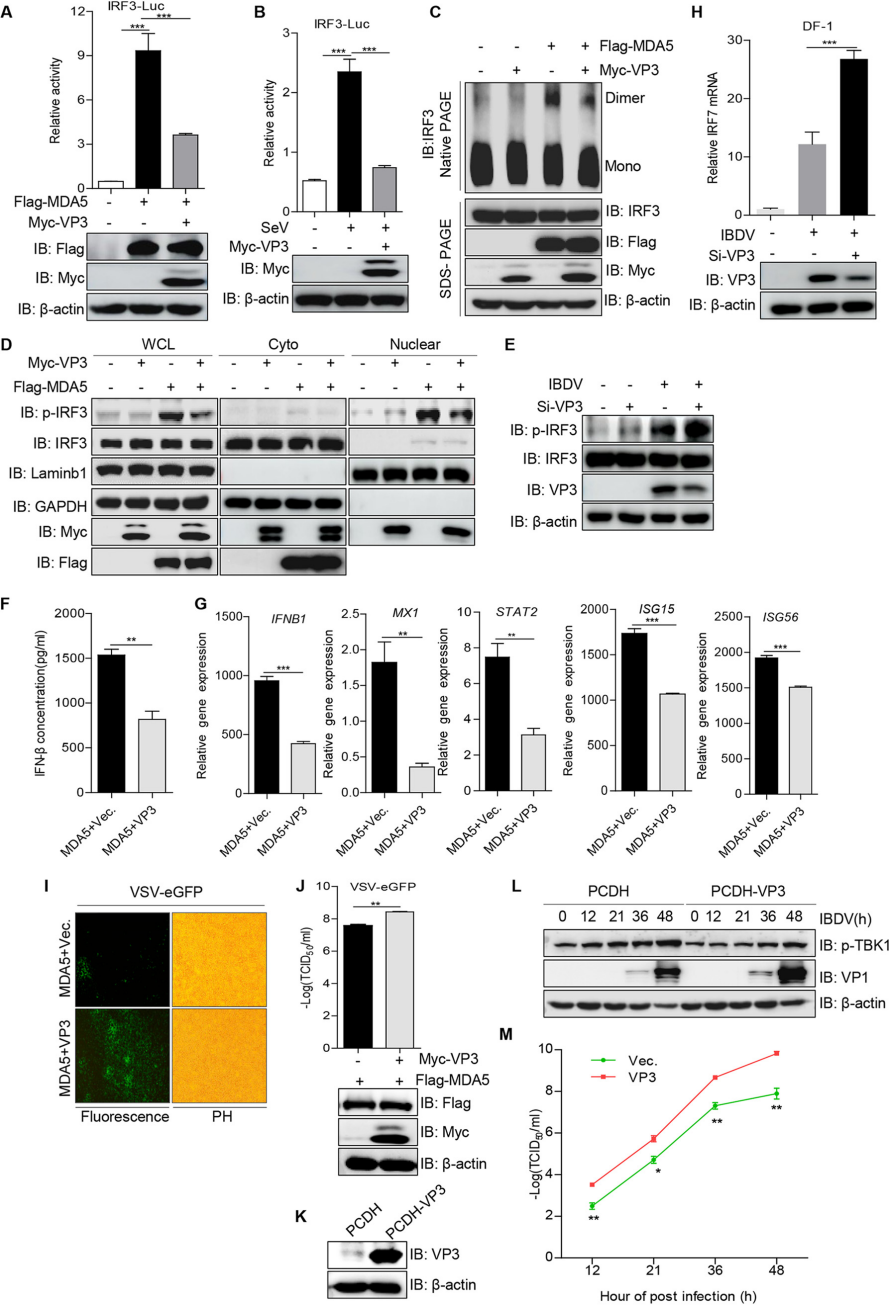
VP3 blocks IFN-β production by inhibiting the activation of IRF3. (A and B) Inhibition of MDA5 (A)- or SeV (B)-mediated IRF3 promoter activation by *Avibirnavirus* VP3. Dual-luciferase assays were performed as described for Fig. 1A and B. (C and D) HEK293T cells were transfected with the expression plasmid of VP3 or an empty vector and with or without MDA5 for 36 h. Cells were then harvested. The levels of IRF3 dimers were subjected to IRF3 dimer gels (C), and the levels of p-IRF3 and total IRF3 in the cytoplasmic and nuclear fractions were subjected to Western blotting (D). GAPDH and LaminB1 were used as the cytoplasmic and nuclear fraction markers, respectively. Mono, monomer; WCL, whole-cell lysates; Cyto, cytoplasm. (E) Impact of VP3 knockdown on IBDV-driven IRF3 activation. HEK293T cells were infected with IBDV at an MOI of 10 for 1 h, and the RNAi sequence (GACGAAGUUGCCAAAGUCUAU) against VP3 was transfected into the resultant cells for another 23 h. The lysates were subjected to an immunoblotting assay with the indicated antibodies. Si-VP3, viral protein VP3 knockdown. (F) Effects of VP3 on MDA5-induced IFN-β production. HEK293T cells were transfected with VP3 or an empty vector along with MDA5 for 36 h. The IFN-β production in response to MDA5 was assessed with an ELISA kit. (G) Effects of VP3 on the transcription of the *IFNB1* and *ISGs* genes. A vector expressing VP3 or an empty vector and MDA5 were transfected into HEK293T cells for 36 h, and then the mRNA levels of the *IFNB1*, *Mx1*, *STAT1*, *ISG15*, and *ISG56* genes were detected by quantitative PCR. The transcript level of each gene was normalized to the expression of *GAPDH*. (H) VP3 knockdown promotes the transcription of chicken *IRF7* induced by IBDV. The method is consistent with that described for Fig. 1F. The mRNA of chicken *IRF7* was detected by qPCR. (I and J) Effects of VP3 on VSV-GFP replication. (I) HEK293T cells were transfected with an empty vector or a vector expressing VP3 protein along with MDA5 for 24 h, cells were infected with VSV-GFP for another 12 h, and virus replication was determined using fluorescence microscopy. PH, phase. (J) The supernatants from VSV-GFP-infected cells were harvested for viral titer detection. (K) Construction of DF-1 cells with stable expression of VP3. (L and M) VP3 overexpression inhibits IFN antiviral signaling in response to IBDV infection. Control and DF1-1 cells overexpressing VP3 were mock infected or IBDV infected. At different time points (0, 12, 21, 36, and 48 h) after IBDV infection, the cellular lysates and the supernatants were subjected to immunoblotting analysis and viral titer detection, respectively. PCDH, empty vector pDCH-CMV-MCS-EF1-Puro. (L and M) Expression levels of the viral structural protein VP1 and p-TBK1 were analyzed (L), and the TCID_50_ was used to assess IBDV replication (M). All data are presented as the means ± SD from three independent experiments. *, *P < *0.05; **, *P < *0.01; ***, *P < *0.001. β-Actin expression served as a loading control.

To confirm the speculation that VP3 can repress IRF3-mediated IFN-β activation, we used the IRF3 reporter to assess the impact of VP3 on IRF3 activation. As shown in Fig. 2A and B, the overexpression of VP3 markedly suppressed the activation of the IRF3 promoter induced by MDA5 or SeV. It is well known that under stimulation conditions, IRF3 is phosphorylated and forms a dimer and then translocates to the nucleus from the cytoplasm for inducing the expression of *IFNB* and the interferon-stimulating gene (ISG) through specifically binding to their promoter regions (25). To obtain the supportive evidence, we detected the effects of VP3 on the MDA5-induced dimerization and nuclear localization of endogenous IRF3. Native PAGE and immunoblotting assays showed that VP3 obviously reduced the activated dimer form of IRF3 driven by MDA5 (Fig. 2C). Similarly, MDA5-induced phosphorylation of IRF3 was greatly suppressed by VP3, and phosphorylated IRF3 was largely distributed in the nucleus (Fig. 2D). Moreover, the phosphorylated IRF3 was increased in IBDV-infected cells with VP3 knockdown by RNAi (Fig. 2E). An enzyme-linked immunosorbent assay (ELISA) of IFN-β also revealed that VP3 significantly decreased IFN-β production (Fig. 2F). In addition, quantitative PCR was used to assess *IFNB* and *ISG* mRNAs, including *MX1*, *STAT2*, *ISG15*, and *ISG56*, triggered by MDA5. As shown in Fig. 2G, VP3 strongly inhibited the transcription of the *IFNB1* and *ISG* genes. All together, these data identify that VP3 decreases IFN-β production via blocking the activation of IRF3 in HEK293T cells. Interestingly, a recent study claimed that chicken IRF3 is IRF7 and that, instead of IRF3, IRF7 is responsible for regulating type I interferon expression (26). Thus, to investigate if VP3 could inhibit IRF7-mediated IFN-β production in chicken, we detected the expression level of *IRF7* mRNA in IBDV-infected DF-1 cells with VP3 knockdown. We observed that the reduction of VP3 facilitated the IBDV-induced transcription of chicken *IRF7* (Fig. 2H), indicating that in chicken, VP3 represses IFN-β expression through an IRF7-mediated pathway.

A recombinant vesicular stomatitis virus (VSV)-green fluorescent protein (GFP) system can be used to screen proteins for IFN-antagonizing activity (27). We employed VSV-GFP to explore whether VP3 protein serves as an antagonist of IFN production. The results showed that VSV-GFP replication was obviously enhanced in HEK293T cells overexpressing VP3 (Fig. 2I and J), suggesting that VP3 has an inhibitory effect on IFN production during RNA virus infection. To test whether VP3 contributes to IBDV proliferation by antagonizing IFN-β production, we constructed a DF-1 cell line with stable expression of VP3 (Fig. 2K). In comparison with that in control cells, phosphorylated TBK1 was obviously decreased in IBDV-infected cells overexpressing VP3, and the viral titer was markedly increased (Fig. 2L and M). Taken together, these findings clearly demonstrate that VP3 protein can support RNA virus replication through suppressing the IRF3-mediated pathway.

### VP3 downregulates IFN-β expression by direct binding of the CC1 domain to TRAF3.

**FIG 3:**
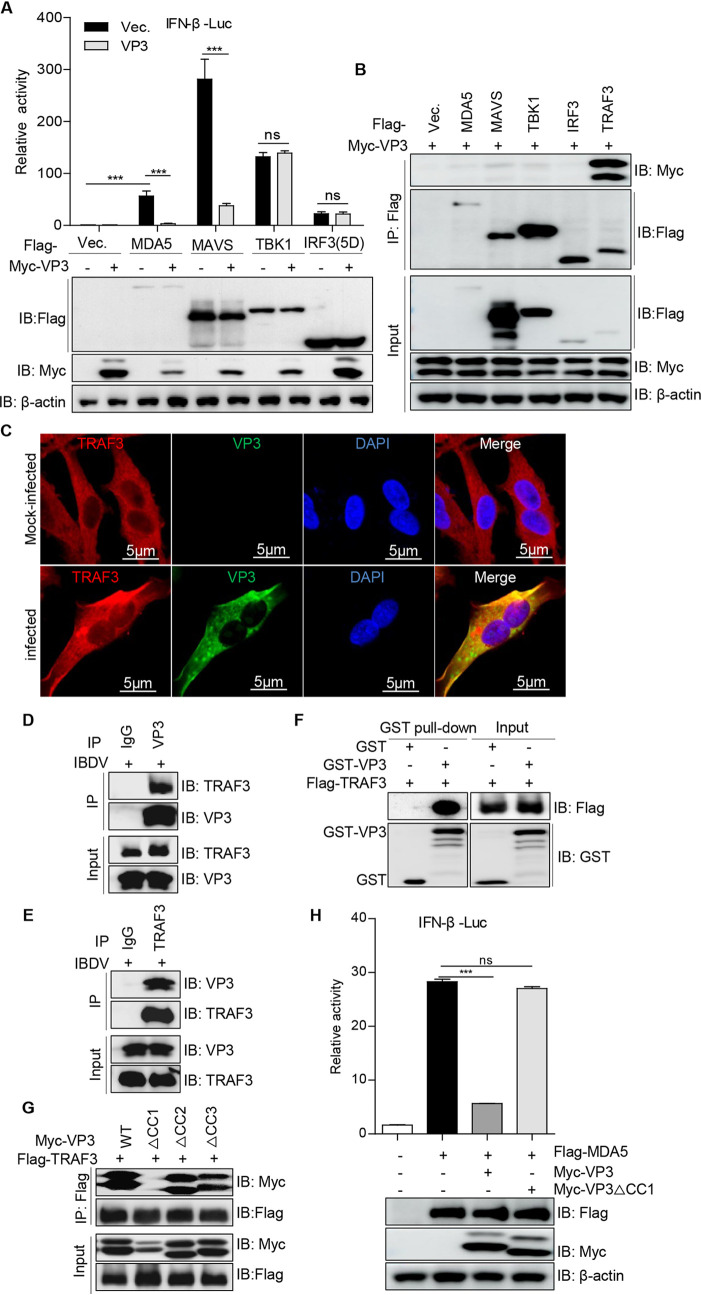
The CC1 domain of VP3 is required for downregulating IFN-β production by interacting with TRAF3. (A) Effects of VP3 on the components of the RLR signaling pathway. HEK293T cells were transfected with the IFN-β reporter, pRL-TK, and an empty vector, MDA5, MAVS, TBK1, or IRF3(5D) along with a control vector or a vector expressing VP3 for 36 h; the IFN-β activities were then measured using a dual-luciferase assay. (B) Identification of the interaction of VP3 with RLR pathway molecules. Flag-tagged MDA5, MAVS, TBK1, IRF3, or TRAF3 and Myc-VP3 were cotransfected into HEK293T cells for 48 h. The lysates were subjected to a Co-IP assay with anti-Flag mouse MAb and immunoblotting with anti-Flag and anti-Myc rabbit PAb. (C) Endogenous TRAF3 colocalization with VP3 during IBDV infection. DF-1 cells were infected with IBDV and were subjected to immunofluorescence staining with anti-TRAF3 rabbit PAb and anti-VP3 mouse MAb. (D and E) *Avibirnavirus* VP3 interacts with endogenous TRAF3 during IBDV infection. HEK293T cells were infected with IBDV (MOI = 10) for 12 h, and cellular lysates were subjected to a Co-IP assay with anti-VP3 mouse MAb (D) or anti-TRAF3 rabbit PAb (E). Anti-IgG mouse or anti-IgG rabbit MAb served as a control. (F) VP3 interacts directly with TRAF3. Purified Flag-TRAF3 was separately mixed with the GST and GST-VP3 proteins, and a GST pulldown assay was conducted as described in Materials and Methods. (G) The CC1 domain of VP3 is responsible for the interaction with TRAF3. HEK293T cells were cotransfected with Myc-tagged VP3, VP3ΔCC1, VP3ΔCC2, or VP3ΔCC3 and Flag-TRAF3. Cellular lysates were subjected to a Co-IP assay with anti-Flag mouse MAb and Western blotting with anti-Flag and anti-Myc rabbit PAbs. (H) The CC1 domain is necessary for VP3 to downregulate IFN-β activation driven by MDA5. HEK293T cells were transfected with the IFN-β reporter, pRL-TK, an empty vector, and an expression plasmid encoding VP3 or VP3△CC1 along with MDA5 for 36 h; luciferase activity was then measured by a dual-luciferase assay.

In order to further investigate in detail the molecular mechanism employed by VP3 to block IRF3-mediated IFN-β production, we detected the impacts of VP3 on IFN-β activation induced by various components in the IRF3 pathway, including MDA5, MAVS, TBK1, and the active form of IRF3 [IRF3(5D)]. Reporter assays showed that VP3 obviously inhibited IFN-β activation triggered by MDA5 or MAVS but did not suppress IFN-β promoter activation driven by TBK1 or IRF3(5D) (Fig. 3A). Thus, we hypothesized that VP3 might inhibit IFN-β activation by targeting MAVS or the TBK1 upstream adaptor TRAF3. A coimmunoprecipitation (Co-IP) assay showed that VP3 strongly associated with TRAF3, but not with MAVS, TBK1, or IRF3 (Fig. 3B), suggesting that TRAF3 is the target of VP3. To validate the relationship between TRAF3 and VP3, an immunofluorescence staining assay was conducted. We observed that viral protein VP3 colocalized with endogenous TRAF3 in IBDV-infected DF-1 cells (Fig. 3C). Moreover, VP3 interaction with endogenous TRAF3 was also detected in IBDV-infected HEK293T cells (Fig. 3D and E). In addition, a glutathione *S*-transferase (GST) pulldown assay verified that VP3 interacts directly with TRAF3 (Fig. 3F). VP3 was demonstrated to contain three coiled-coil (CC) domains, CC1, CC2, and CC3 (28). Binding domain mapping showed that the CC1 domain of VP3 could interact with TRAF3 (Fig. 3G). Then, to determine whether this domain was necessary for VP3 to negatively regulate IFN-β production, the activity of the IFN-β promoter driven by MDA5 was detected. A reporter assay showed that VP3 strongly decreased MDA5-driven IFN-β promoter activation (Fig. 3H), indicating that the CC1 domain of VP3 plays a crucial role in blocking MDA5-mediated IFN-β induction.

### The residue lysine-155 of TRAF3 is essential for VP3 to downregulate IFN-β production.

**FIG 4:**
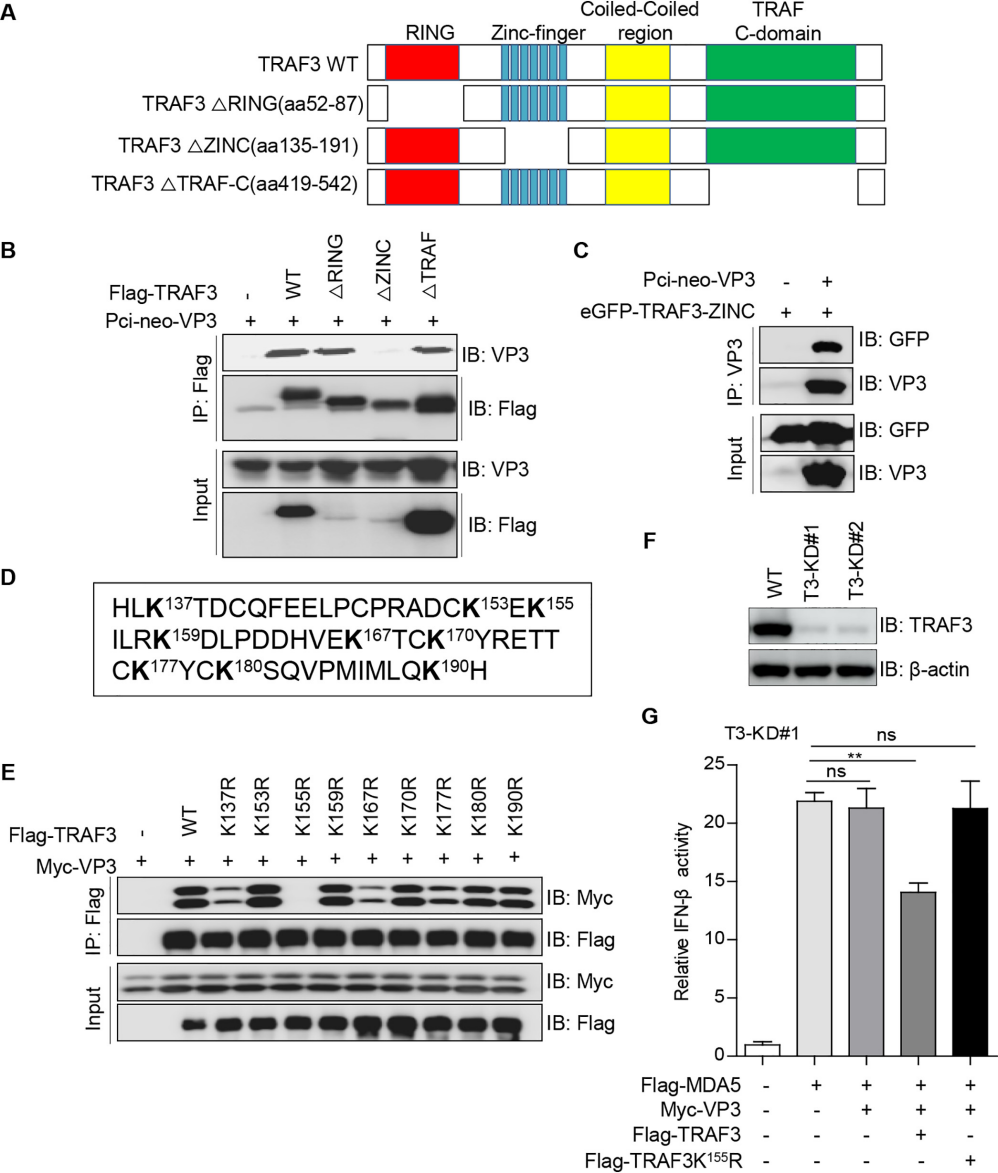
The residue lysine-155 of TRAF3 is necessary for VP3 to downregulate IFN-β production. (A) Schematic representation of different domains of TRAF3 and its deletion mutants. aa, amino acids. (B) Identification of the TRAF3 domain binding to *Avibirnavirus* VP3. HEK293T cells were cotransfected with VP3 and Flag-tagged TRAF3, TRAF3△RING, TRAF3△zinc finger, or TRAF3△TRAF-C for 48 h. Cellular lysates were subjected to a Co-IP assay with anti-Flag mouse MAb and an immunoblotting assay using anti-Flag and anti-Myc rabbit PAbs. (C) The zinc finger domain of TRAF3 is essential for the interaction with VP3. A zinc finger fragment was cloned into the eGFP-C3 vector, and then the resulting construct and vector encoding *Avibirnavirus* VP3 were cotransfected into HEK293T cells for 48 h. Cellular lysates were subjected to a Co-IP assay with anti-VP3 mouse MAb and an immunoblotting assay with anti-GFP and anti-VP3 mouse MAbs. (D) Residual sequence of the zinc finger domain of TRAF3. The lysine residues are displayed in boldface. (E) The residue lysine-155 of TRAF3 is crucial for the interaction with VP3. Different Flag-TRAF3 mutants bearing a single lysine (K)-to-arginine (R) substitution and Myc-VP3 were cotransfected into HEK293T cells for 48 h. Cellular lysates were subjected to a Co-IP assay using anti-Flag mouse MAb and an immunoblotting assay with anti-Flag and anti-Myc rabbit PAbs. (F) *TRAF3*-deficient HEK293T cells were generated by the CRISPR-Cas9 method. (G) Effects of the residue lysine-155 of TRAF3 on the inhibition of MDA5-mediated IFN-β activation by VP3. *TRAF3*-deficient HEK293T cells were transfected with the IFN-β reporter and pRL-TK, along with an empty vector or a vector expressing VP3 together with MDA5 or MDA5 and TRAF3-WT or TRAF3-K155R for 36 h. The luciferase activities were assessed by a dual-luciferase assay. All data are presented as the means ± SD from three independent experiments. ns, *P* > 0.05; **, *P < *0.01.

To identify the binding domain of TRAF3 responsible for the interaction with *Avibirnavirus* VP3, we constructed a series of TRAF3 truncation mutants (Fig. 4A). Co-IP assays showed that the zinc finger domain of TRAF3, but not its RING and TRAF-C domains, is responsible for the association with *Avibirnavirus* VP3 (Fig. 4B). To further confirm this result, the expression plasmid encoding the zinc finger domain of TRAF3 was constructed. In a Co-IP assay, we found that the zinc finger domain of TRAF3 interacted with *Avibirnavirus* VP3 (Fig. 4C). A previous report showed that the TRAF3-binding partner acts not simply through the recognition of bound TRAF3 molecules but also through the detection of the ubiquitin-modified form of TRAF3 (29). Ubiquitin was conjugated to the lysine (K) residue of substrates (30). Thus, to investigate if TRAF3’s association with VP3 is TRAF3 ubiquitination dependent, we replaced every lysine residue within the zinc finger domain of TRAF3 with arginine (R) (Fig. 4D). A Co-IP assay showed that a single amino acid substitution at lysine-155 of TRAF3 was sufficient to abolish TRAF3’s strong interaction with VP3 (Fig. 4E), suggesting that lysine-155 of TRAF3 may be ubiquitylated, which is responsible for the interaction with VP3. To probe whether the inhibitory effect of VP3 on MDA5-driven IFN-β activation depends on lysine-155 of TRAF3, *TRAF3*-deficent HEK293T cells were generated by the clustered regularly interspaced short palindromic repeat (CRISPR)-Cas9 method (Fig. 4F). An IFN-β luciferase reporter assay showed that the inhibitory effect of VP3 on MDA5-mediated IFN-β activation was not obviously present in *TRAF3*-deficient HEK293T cells (Fig. 4G), which further indicated that TRAF3 participates in VP3’s negative regulation of the IFN-β signaling pathway. Moreover, we discovered that the inhibitory effect could obviously be rescued in TRAF3-deficient HEK293T cells overexpressing wild-type TRAF3 (TRAF3-WT), but not in those overexpressing the TRAF3 K^155^R mutant (Fig. 4G). Taken together, our data reveal that the residue lysine-155 of TRAF3 is necessary for VP3 to inhibit IFN-β production triggered by MDA5.

### *Avibirnavirus* VP3 blocks the formation of the TRAF3/TBK1 complex by repressing K33-linked polyubiquitination of the residue lysine-155 of TRAF3.

**FIG 5:**
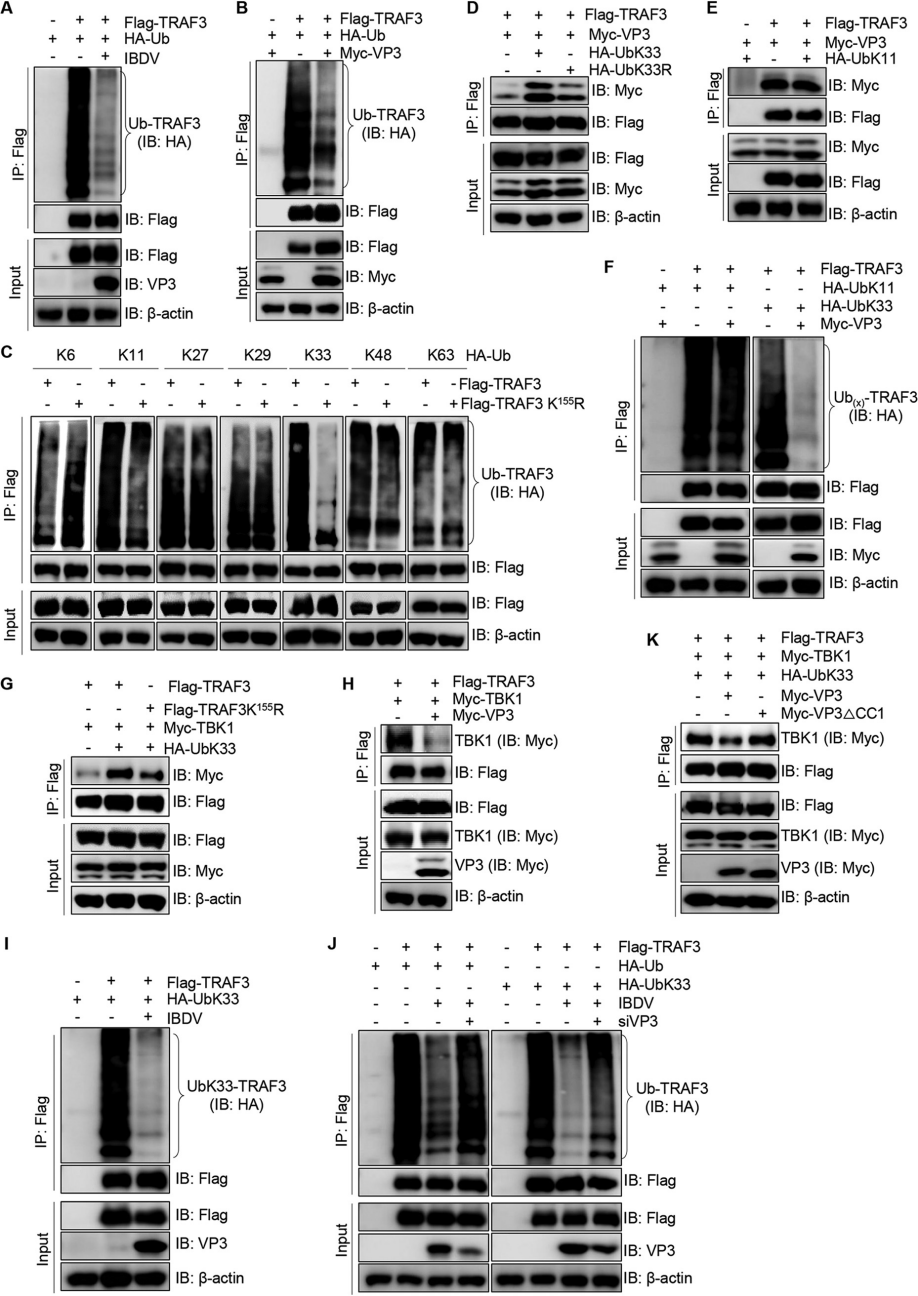
VP3 decreases the interaction of TRAF3 with TBK1 by inhibiting K33-linked polyubiquitination of the residue lysine-155 of TRAF3. (A) IBDV infection suppresses the ubiquitination of TRAF3. HEK293T cells were cotransfected with Flag-TRAF3 and HA-Ub or the corresponding empty vector for 24 h. The resultant cells were infected with IBDV for another 24 h. The lysates were subjected to immunoprecipitation and Western blotting assays with the indicated antibodies. (B) Viral protein VP3 represses TRAF3 ubiquitination. HEK293T cells were cotransfected with HA-Ub and Flag-TRAF3 along with an empty vector or Myc-VP3 for 48 h. Cellular lysates were subjected to a ubiquitination assay with anti-Flag mouse MAb and an immunoblotting assay using anti-HA mouse MAb and anti-Flag and anti-Myc rabbit PAbs. (C) Identification of the ubiquitination phenotype of the residue lysine-155 of TRAF3. Flag-TRAF3-WT or Flag-TRAF3-K155R and different HA-Ub mutants were cotransfected into HEK293T cells for 48 h. Cell lysates were subjected to a ubiquitination assay with anti-Flag mouse MAb and a Western blotting assay using anti-Flag rabbit PAb and anti-HA mouse MAb. (D) K33-linked ubiquitin chains enhance the association of TRAF3 and VP3. HEK293T cells were cotransfected with Flag-TRAF3 and Myc-VP3 along with HA-UbK33 or HA-UbK33R for 48 h. Cellular lysates were subjected to Flag precipitation and an immunoblotting assay with anti-Flag and anti-Myc PAbs. (E) The association between TRAF3 and VP3 is not affected by K11-linked polyubiquitin chains. Flag-TRAF3, Myc-VP3, and HA-UbK11 or an empty vector were cotransfected into HEK293T cells for 48 h. Cellular lysates were immunoprecipitated with anti-Flag mouse MAb, followed by immunoblotting using the indicated antibodies. (F) Impact of VP3 overexpression on TRAF3 K11- and K33-linked polyubiquitination. HEK293T cells were cotransfected with Flag-TRAF3, HA-UbK11, or HA-UbK33 and Myc-VP3 or the corresponding empty vector for 48 h. The lysates were subjected to immunoprecipitation and Western blotting assays using the indicated antibodies. (G) K33-linked polyubiquitination of the residue lysine-155 of TRAF3 benefits the interaction with TBK1. Flag-TRAF3-WT or a Flag-TRAF3-K155R mutant and Myc-TBK1 with or without HA-UbK33 were separately cotransfected into HEK293T cells for 48 h. Cellular lysates were subjected to a Co-IP assay with anti-Flag mouse MAb and an immunoblotting assay with anti-Flag and anti-Myc rabbit PAbs. (H) VP3 reduces the interaction of TRAF3 and TBK1. HEK293T cells were cotransfected with Flag-TRAF3 and Myc-TBK1 along with an empty vector or a vector expressing VP3 for 48 h. Cellular lysates were subjected to a Co-IP assay using anti-Flag mouse MAb and a Western blotting assay using anti-Flag and anti-Myc rabbit PAbs. (I) IBDV infection suppresses TRAF3 K33-linked polyubiquitination. HEK293T cells were cotransfected with Flag-TRAF3 and HA-UbK33 or the corresponding empty vector for 24 h, followed by infection with IBDV for another 24 h. The lysates were subjected to immunoprecipitation and a Western blotting assay with the indicated antibodies. (J) VP3 knockdown greatly blocks the decrease of IBDV-induced TRAF3 polyubiquitination. HEK293T cells were cotransfected with Flag-TRAF3 and HA-Ub or HA-UbK33 for 24 h, followed by infection with IBDV. At 1 h after infection, the resultant cells were transfected with the RNAi sequence against VP3 for another 23 h. Cellular lysates were subjected to immunoprecipitation and immunoblotting assays using the indicated antibodies. (K) The CC1 domain is required for VP3 to decrease the association of TRAF3 with TBK1. Flag-TRAF3, Myc-TBK1, and HA-UbK33 along with an empty vector, an expression plasmid encoding VP3 or VP3△CC1, were cotransfected into HEK293T cells for 48 h, respectively. Cellular lysates were subjected to a Co-IP assay with anti-Flag mouse MAb and an immunoblotting assay with anti-Flag and anti-Myc rabbit PAbs.

TRAF3 undergoes polyubiquitination to provide a scaffold for complex formation, which plays a crucial role in the host antiviral innate immune response (31). Thus, to investigate the impact of VP3 on TRAF3 ubiquitination, a ubiquitination assay was conducted. We found that overexpression of viral protein VP3 and *Avibirnavirus* infection significantly decreased TRAF3 ubiquitination (Fig. 5A and B). Given the result in Fig. 4E, we hypothesized that lysine-155 of TRAF3 might be ubiquitylated. Thus, to explore the type of polyubiquitination of lysine-155 of TRAF3, we constructed a series of ubiquitin mutants. A ubiquitination assay showed that K11- and K33-linked polyubiquitination of the TRAF3 K^155^R mutant were significantly decreased compared with that of TRAF3-WT (Fig. 5C), suggesting that the lysine-155 residue of TRAF3 may be conjugated by K33- and K11-linked polyubiquitin chains. Considering the result that lysine-155 of TRAF3 is essential for the interaction with *Avibirnavirus* VP3 (Fig. 4E), we hypothesized that K11- and K33-ubiquitin might affect the interaction of TRAF3 and VP3. As shown in Fig. 5D, the interaction between TRAF3 and VP3 was enhanced in KEK293T cells overexpressing K33 ubiquitin, and the TRAF3 interaction with VP3 was significantly decreased in HEK293T cells overexpressing K33R ubiquitin compared with that in HEK293T cells overexpressing K33 ubiquitin. However, the TRAF3 association with VP3 is not obviously altered in K11-ubiquitin-overexpressing cells (Fig. 5E), and VP3 overexpression significantly inhibited the K33-linked polyubiquitination of TRAF3 but not its K11-linked polyubiquitination (Fig. 5F). These results indicated that VP3 may recognize the K33-linked ubiquitin-modified form of TRAF3.

The formation of the TBK1-TRAF3 complex directs IRF3 phosphorylation, which is required for transcription from the IFN-beta promoter (5, 32). To explore the effects of K33-linked polyubiquitination of TRAF3 on the IFN signaling pathway, we detected the interaction of WT and K155R mutant TRAF3 and its downstream signal molecule TBK1 in HEK293T cells with or without K33 ubiquitin overexpression. As shown in Fig. 5G, K33 ubiquitin overexpression obviously enhanced the association of TRAF3 and TBK1. Meanwhile, we observed that the increase of the interaction of TRAF3 and TBK1 by K33 ubiquitin was reduced when the lysine-155 residue was replaced with arginine (Fig. 5G), suggesting that K33 polyubiquitination of lysine-155 of TRAF3 is beneficial to TBK1-TRAF3 complex formation and may act as a positive regulator of the IFN signaling pathway. Given that VP3 binds to K33-linked ubiquitinated TRAF3 and inhibits its ubiquitination (Fig. 4E and 5B and F), we speculated that the inhibitory effect of VP3 on IFN-β induction was relevant in interfering with the formation of the TRAF3-TBK1 complex by reducing the K33-linked polyubiquitination of lysine-155 of TRAF3. As shown in Fig. 5H, in a Co-IP assay, the interaction of TRAF3 and TBK1 was dramatically reduced in VP3-overexpressing cells. Meanwhile, the K33-linked polyubiquitination of TRAF3 was dramatically reduced in IBDV-infected cells (Fig. 5I). Subsequently, to investigate the role of VP3 on regulating TRAF3 ubiquitination during IBDV infection, RNAi against viral protein VP3 was used. Data shown in Fig. 5J revealed that VP3 knockdown greatly decreased the reduction of IBDV-induced TRAF3 ubiquitination. In addition, we found that the CC1 domain was required for VP3 to reduce the interaction of TRAF3 and TBK1 (Fig. 5K), which further indicated that the CC1 domain plays an important role in the negative regulation of VP3 in IFN-β production. Taken together, these findings reveal that the physical association of VP3 with K33-linked ubiquitinated TRAF3 decreases TRAF3-TBK1 complex formation to block IFN signaling by inhibiting K33 polyubiquitination of the residue lysine-155 of TRAF3.

## DISCUSSION

Given the importance of the RLR-induced IFN signaling pathway, it is not surprising that many viruses have evolved diverse and elaborate mechanisms that target the components of RLR pathways to inhibit IFN-β production for supporting self-replication. In the past decade, growing evidence has demonstrated that viral proteins have adopted multiple strategies to evade the host IFN response. Porcine delta coronavirus accessory protein NS6 antagonizes IFN-β production by interfering with the binding of RIG-I/MDA5 to double-stranded RNA (33). The influenza virus protein PB1-F2 inhibits the induction of type I interferon at the level of MAVS (34). NS1 protein of influenza A virus (IAV) not only targets RIG-I but also associates with TRAF3 to inhibit IFN-β induction (35–38). Additionally, studies have demonstrated that multifunctional protein VP3 of *Avibirnavirus* inhibits the host antiviral response and subsequently promotes viral replication and pathogenesis (28, 39–41). Recently, it has been reported that VP3 can compete with MDA5 to bind the intracellular viral genomic dsRNA to inhibit IFN-β production (11). In the present study, we found that *Avibirnavirus* VP3 could also inhibit MDA5-driven IFN-β activation, not RIG-I (Fig. 1A and B). This observation is in agreement with the fact that the chicken genome does not contain the RIG-I gene (42). Moreover, in recent years, studies have further demonstrated that chicken RIG-I is missing (43) and that MDA5 possesses the same structure as and functionally compensates for the deficiency of RIG-I (44, 45). Actually, our study showed that *Avibirnavirus* VP3 suppressed the activation of IFN-β at the level of the TRAF3 adaptor, providing new insight into the mechanism used by VP3 to antagonize the production of IFN-β. IFN-β activation required the cooperative activation of IRF3 and NF-κB (24). We observed that VP3 could inhibit IFN-β promoter activation by disrupting the activation of IRF3 and NF-κB in HEK293T cells. However, in the present study, we exhibited in detail only the molecular mechanism of how VP3 downregulates IRF3-mediated IFN-β production; the details of the mechanism that VP3 uses to negatively modulate NF-κB-mediated IFN-β production are not shown but will be shown in another paper.

Ubiquitination is a reversible posttranslational modification that plays a crucial role in regulating host IFN-I signaling (46–48). During RNA virus infection, TRAF3 forms different types of ubiquitination, according to distinct subsets of innate signaling events that lead to distinct outcomes. At the early stage of virus infection, K63-linked polyubiquitination of TRAF3 contributes to the formation of the IFN antiviral signaling complex for promoting IFN induction; however, at the late stage of virus infection, K48-linked polyubiquitination of TRAF3 mediated by TRIAD3A facilitates its degradation through the ubiquitin proteasome pathway (20, 49). Thus, many viruses have also employed multiple strategies to regulate the ubiquitination of TRAF3 for supporting viral replication (50–53). A recent report demonstrated that K33-linked polyubiquitination of TRAF3 orchestrated the expulsion of intracellular bacteria from the TLR4 pathway (29). However, the roles of K33 polyubiquitination of TRAF3 in IFN-β induction are not known. In the present study, we revealed that the residue lysine-155 of TRAF3 was the key K33-linked ubiquitination site and that it is necessary for the interaction with the *Avibirnavirus* VP3 protein. Upon virus infection, K63-linked polyubiquitination of TRAF3 leads to the recruitment of TBK1 and the subsequent activation of IRF3 (54–56). However, in the present study, we surprisingly discovered that the K33-linked polyubiquitination of the residue lysine-155 of TRAF3 also contributed to the interaction with TBK1, and the inhibitory effect of VP3 on IFN-β induction is also closely relevant to the K33-linked polyubiquitination of lysine-155 of TRAF3. Mechanistically, VP3 suppressed the formation of the TRAF3-TBK1 complex through decreasing K33-linked polyubiquitination of lysine-155 of TRAF3, which resulted in a blockage of IFN signaling. Furthermore, we identified that the CC1 domain plays a crucial role in the negative modulation of VP3 in IFN-β production.

In conclusion, we first identified that K33-linked polyubiquitination of the residue lysine-155 of TRAF3 is beneficial to its interaction with TBK1, which may positively regulate the host IFN immune response. Next, we found that *Avibirnavirus* VP3 negatively regulates the K33-linked polyubiquitination of TRAF3 and that VP3’s interaction with TRAF3 disrupts the formation of the TRAF3-TBK1 complex, which contributes to the downregulation of host IFN signaling, supporting viral replication. These findings not only provide new insight into the role of the ubiquitination of TRAF3 in IFN signaling but also increase our understanding of the mechanism that allows *Avibirnavirus* VP3 to evade the host innate antiviral immune response.

## MATERIALS AND METHODS

### Cells and virus.

DF-1 cells (CRL-12203 chicken embryo fibroblasts; American Type Culture Collection [ATCC], Manassas, VA, USA) and HEK293T cells (CRL-11268 human embryonic kidney cells; ATCC) were routinely cultured in Dulbecco’s modified Eagle’s medium (DMEM; Gibco, Carlsbad, CA) supplemented 10% fetal bovine serum (FBS). IBDV stain NB, SeV, and recombinant vesicular stomatitis virus (VSV-GFP) were stored in our laboratory and have been described previously (57, 58).

### Antibodies and reagents.

Anti-Flag (F1804-5mg), anti-Myc (05-419), and anti-hemagglutinin (HA; H9658) mouse monoclonal antibodies (MAbs) were purchased from Sigma-Aldrich (St. Louis, MO, USA). Anti-Myc (R1208-1) and anti-Flag (0912-1) rabbit polyclonal antibodies (PAbs), anti-lamin B1 (ET1606-27) rabbit MAb, and anti-β-actin (EM21002) mouse MAb were purchased from HuaAn Biotechnology (Hangzhou, China). Anti-TRAF3 (D260776-0100) rabbit PAb for Western blotting (WB) was purchased from Sangon Biotech (Shanghai, China). Mouse MAbs against IBDV proteins (VP1, VP2, VP3, VP4, and VP5) were prepared in our laboratory (59). Anti-IRF3 (11312-1-AP) rabbit PAb was purchased from Proteintech (Wuhan, China). Phospho-IRF3 (Ser386) (number 4947) and phospho-TBK1 (number 5483) rabbit MAbs were purchased from Cell Signaling Technology (Danvers, MA, USA). A dual-luciferase reporter gene assay kit (RG027) was purchased from Beyotime (Shanghai, China). Exfect transfection reagent (T101-01/02) for plasmids was purchased from Vazyme Biotechnology (Nanjing, China). jetPRIME transfection reagent (PT-114-15; Polyplus, New York, NY, USA) was used for RNAi transfection. A human IFN-β ELISA kit (JL19215) was purchased from Jianglai Biotechnology (Shanghai, China). Puromycin (ant-pr-1), poly(I·C) (tlrl-pic), and VACV-70 (tlrl-vav70n) were purchased from InvivoGen (San Diego, CA).

### Constructs.

IFN-β, IRF3, and ISRE promoter luciferase reporter plasmids were kindly provided by Jihui Ping from the Nanjing Agricultural University and from Zongping Xia of Zhejiang University. The mammalian expression plasmids Flag-tagged RIG-I, MDA5, MAVS, TBK1, IRF3, and constitutively active IRF3 [IRF3(5D)] were stored in our laboratory. TRAF3 (NCBI accession no. XM_004936343.3) was amplified from DF-1 cells and subcloned into the pCMV-Flag-N (635688; Clontech) vector. TRAF3 or Ub mutants were constructed by a standard mutagenesis method. Enhanced GFP (eGFP)-TRAF3-zinc was constructed by inserting a TRAF3-zinc fragment into the eGFP-C3 empty vector. pGEX-4T-VP3, Myc-tagged VP3, and its truncated mutants have been described previously (28). The primers used for cloning are listed in Table 1.

**TABLE 1:** Primers used for cloning and quantitative real-time PCR

Name	Forward sequence (5′–3′)^*a*^	Reverse sequence (5′–3′)^*a*^
*TRAF3*	gg *GAATTC*GCATGGACA	gg *GTCGAC*TCAGGGGTC
	CCAGTAAGAAGACAGAAC	TGGTAGATCCGATGTA
*TRAF3*△RING	GAGGATAAGTATAAAA	CTTTCTTGACATGCT
	CAGCATGTCAAGAAAG	GTTTTATACTTATCCTC
*TRAF3*△Zinc	CTTGGGTCAATTACTGATG	CACACGGGCAGTCTGTAT
	GAAGATACAGACTGCCCG	CTTCCATCAGTAATTGACC
*TRAF3*△TRAF	CAATGGAGTGTTAATTGAG	CTTTAATATACGTTCCATT
	AATGGAACGTATATTAAAG	CTCAATTAACACTCCATTG
*TRAF3* K^137^R	CAATTACTGATGCATTT	CTGACAATCAGTTCTCA
	GAGAACTGATTGTCAG	AATGCATCAGTAATTG
*TRAF3* K^153^R	CCTCGTGCTGATTGTAG	CTCAGTATTTTTTCTC
	AGAAAAAATACTGAG	TACAATCAGCACGAGG
*TRAF3* K^155^R	CTGATTGTAAAGAAA	CTTTTCTCAGTATTC
	GAATACTGAGAAAAG	TTTCTTTACAATCAG
*TRAF3* K^159^R	GAAAAAATACTGAGAA	GTGGTCTGGCAAATCT
	GAGATTTGCCAGACCAC	CTTCTCAGTATTTTTTC
*TRAF3* K^167^R	GCCAGACCACGTAGAGA	CGGTATTTACAGGTCC
	GGACCTGTAAATACCG	TCTCTACGTGGTCTGGC
*TRAF3* K^170^R	CGTAGAGAAGACCTGT	GTTGTCTCTCGGTATC
	AGATACCGAGAGACAAC	TACAGGTCTTCTCTACG
*TRAF3* K^177^R	CGAGAGACAACTTGTAG	CTTGGCTTTTACAGTAT
	ATACTGTAAAAGCCAAG	CTACAAGTTGTCTCTCG
*TRAF3* K^180^R	CTTGTAAATACTGTAG	CATTGGGACTTGGCT
	AAGCCAAGTCCCAATG	TCTACAGTATTTACAAG
*TRAF3* K^190^R	CAATGATTATGTTGCAG	CTGTATCTTCATGTCT
	AGACATGAAGATACAG	CTGCAACATAATCATTG
*Ub*K6	GCAGATCTTCGTGAA	CTACCAGTCAGGGTC
	GACCCTGACTGGTAG	TTCACGAAGATCTGC
*Ub*K11	AGGACCCTGACTGGT	CGAGAGTGATGGTCT
	AAGACCATCACTCTCG	TACCAGTCAGGGTCCT
*Ub*K27	CACCATTGAGAATGTC	CTTGGATCCTTGCCTT
	AAGGCAAGGATCCAAG	GACATTCTCAATGGTG
*Ub*K29	GAGAATGTCAGGGCA	CCTGTCTTGGATCTT
	AAGATCCAAGACAGG	TGCCCTGACATTCTC
*Ub*K33	CAAGGATCCAAGACA	GGAGGGATGCCTTCC
	AGGAAGGCATCCCTCC	TTGTCTTGGATCCTTG
*Ub*K33R	GCAAAGATCCAAGACA	GGAGGGATGCCTTCC
	GGGAAGGCATCCCTCC	CTGTCTTGGATCTTTGC
q*IFN-β*	TGAACTCCACCAGCAGACAG	CCACCATCCAGGCGTAGC
q*ISG15*	CCTGGTGTCCGTGACTAACTC	AGACCTCATAGATGTTGCTGTGG
q*ISG56*	TGTGCTGAGATGGACTGTGAG	GGCGATAGGCTACGACTGC
q*MX1*	ACCTCGTGTTCCAACTGAAG	GTGTGATGAGCTCGCTGGTA
q*STAT2*	CTGCTAGGCCGATTAACTACCC	TCTGATGCAGGCTTTTTGCTG
q*GAPDH*	ATGACATCAAGAAGGTGGTG	CATACCAGGAAATGAGCTTG
qch*IFN-β*	ACCAGGATGCCAACTTCTCTTGGA	ATGGCTGCTTGCTTCTTGTCCTTG
qch*IRF7*	ACCACATGCAGACAGACTGACACT	GGAGTGGATGCAAATGCTGCTCTT
qch*GAPDH*	CCAGCAACATCAAATGGGCAGAT	TGATAACACGCTTAGCACCACCCT

a gg, restriction endonuclease protective base; italics, restriction endonuclease sequence.

### Reporter assay.

HEK293T cells were seeded onto 24-well plates and transfected on the following day with Exfect transfection reagent. Reporter plasmids (IFN-β-Luc, ISRE-Luc, or IRF3-Luc) and pRL-TK (Promega; E2241) were cotransfected along with the indicated plasmids. At the indicated times after transfection, cells were treated with different stimulators. Luciferase activity was detected using the dual-luciferase assay kit according to the manufacturers’ instructions. All experiments were performed in triplicate.

### RNA extraction and qPCR.

RNA was extracted using TRIzol reagent according to the manufacturer’s instructions. One microgram of RNA was then reverse transcribed with a PrimeScript reverse transcriptase (RT) reagent kit with gDNA Eraser (TaKaRa, Kusatsu, Japan). Quantitative real-time PCR (qPCR) was performed with TB Green Premix *Ex Taq* (TaKaRa) on a LightCycler 96 system (Roche). *GAPDH* was applied to normalize the relative abundances of the indicated gene mRNAs. The primers used for qPCR are listed in Table 1.

### Recombinant protein purification.

Escherichia coli BL21(pLysS) cells harboring the pGEX-4T-1-VP3 plasmid were cultured separately in 200 ml of Luria-Bertani (LB) medium and induced with 1 mM isopropyl β-d-thiogalactopyranoside (IPTG; Sangon Biotech, Shanghai, China) at 16°C, with shaking at 90 rpm, overnight. Cell pellets were lysed by sonication in the binding buffer (50 mM Tris-Cl, 150 mM NaCl, pH 8.0). After centrifugation at 12, 000 × *g* for 10 min, the supernatant was incubated with GST resin for 4 h at 4°C, and then the beads were washed with ice-cold binding buffer. Finally, the protein was eluted in binding buffer containing 2 mg/ml reduced glutathione (A100399-0005; Sangon Biotech). For Flag-TRAF3 protein, HEK293T cells were transfected with Flag-TRAF3 for 24 h. Cells were lysed with NP-40 lysis buffer (P0013F; Beyotime, China) containing phenylmethylsulfonyl fluoride (PMSF). After centrifugation at 12,000 × *g* for 10 min, the supernatant was incubated with anti-FLAG M2 affinity gel (A2220; Sigma-Aldrich) for 4 h at 4°C. The beads were washed five times with ice-cold phosphate-buffered saline (PBS) at 4°C. Finally, the protein was eluted with 0.5 mg/ml 3*FLAG peptide (A6001; APExBIO, Houston, TX, USA).

### Western blotting.

Cellular lysates or immunoprecipitation complexes were separated by SDS-PAGE and transferred to a nitrocellulose blotting membrane (10600001; GE Healthcare Life Science). After being blocked in 5% skim milk containing 0.1% Tween 20 for 1 h at room temperature, the membranes were washed and then incubated with the indicated primary antibodies at 4°C overnight and then with horseradish peroxidase-conjugated goat anti-mouse/rabbit IgG. Finally, protein bands were visualized using enhanced chemiluminescence (ECL) and imaged using AI680 Images (GE Healthcare, USA).

### Co-IP, ubiquitination, and GST pulldown assays.

For Co-IP and ubiquitination assays, HEK293T cells transfected with the indicated plasmids or undergoing specific treatment were lysed in NP-40 lysis buffer containing PMSF at 4°C for 4 h. After centrifugation at 12,000 × *g* for 10 min, the supernatant was incubated with the indicated antibody and protein A/G PLUS-agarose (sc-2003; Santa Cruz Biotechnology, Santa Cruz, CA, USA) at 4°C for 4 h. For GST pulldown assay, purified GST, and GST-VP3 protein were separately mixed with purified Flag-TRAF3 protein and GST resin (L00206; GenScript, Nanjing, China) at 4°C for 4 h. After centrifugation, the supernatant was discarded, and the pellets were washed five times with NP-40 lysis buffer. Finally, the pellet was lysed in lysis buffer for an immunoblotting assay.

### ELISA for IFN-β.

The cultured media were collected for the measurement of IFN-β using a human IFN-β ELISA kit according to the manufacturer’s instructions.

### Immunofluorescent (IF) staining.

DF-1 cells were seed on the 35-mm glass bottom cell culture dishes and infected on the following day with IBDV. At 12 h after infection, the cells were fixed with 4% paraformaldehyde for 10 min at room temperature and permeabilized for 5 min with 0.2% Triton X-100. After being blocked with 5% skim milk, cells were incubated with anti-VP3 mouse MAb and anti-TRAF3 rabbit PAb (A15106; ABclonal Technology, Wuhan, China) overnight at 4°C. After being washed three times with PBS, cells were further incubated with fluorescein isothiocyanate (FITC)-labeled goat anti-mouse IgG and Alexa Fluor 546-labeled donkey anti-rabbit IgG secondary antibody at room temperature for 1 h. Cellular nuclei were stained with DAPI (4′,6-diamidino-2-phenylindole) for 10 min and viewed with an LSM780 laser scanning confocal microscope (Zeiss, Oberkochen, Germany).

### IRF3 dimer gels.

HEK293T cells were seeded on 6-well plates and transfected with the indicated plasmids on the following day. At 36 h after transfection, cells were collected, resuspended in gentle radioimmunoprecipitation assay (RIPA) lysis buffer (P0013D; Beyotime) containing PMSF, and kept overnight at −80°C. After being freeze-thawed three times, the lysates were centrifuged at 12,000 × *g* for 10 min, and the supernatant was then divided into two parts. One portion was added with the native sample buffer (1 M pH 6.8 Tris-HCl, 0.02% bromophenol blue, 50% glycerol) and subjected to an analysis of IRF3 dimers. IRF3 dimer gels were electrophoresed as described previously (60). Briefly, a native PAGE gel was prerun with 25 mM Tris-HCl and 192 mM glycine, pH 8.4, with and without 1% deoxycholate (DOC) in the cathode and anode chambers, respectively, for 30 min at 70 V. Samples in the native sample buffer were applied to the gel and electrophoresed for another 3 h at 70 V. Protein bands separated by native PAGE were transferred to a nitrocellulose blotting membrane using transfer buffer (25 mM Tris-HCl and 192 mM glycine, 20% methanol). The subsequent procedures were the same as for Western blotting. The other portion was added to SDS sample buffer for Western blotting using anti-IRF3 rabbit PAb. β-Actin expression served as a loading control.

### Cellular fractionation.

We conducted cellular fractionation as described previously (61). Briefly, HEK293T cells were transfected with the indicated plasmids for 48 h. Cells were lysed in 0.1% NP-40 lysis buffer (Beyotime) diluted with PBS. After centrifugation at 1,000 × *g* for 5 min, the supernatant was transferred into a new 1.5-ml tube and subjected to another round of centrifugation (15,000 × *g* for 10 min, 4°C). The clear supernatant was collected as the cytoplasmic fraction. The pellet was washed in ice-cold 0.1% NP-40 lysis buffer two times. The supernatant was discarded, and the pellet was kept as the nuclear fraction.

### Generation of DF-1 cells with stable expression of VP3.

The VP3 fragment was inserted into the lentivirus vector PCDH-CMV-MCS-EF1-Puro (System Biosciences; CD510B-1). The recombinant construct and ViraPower lentiviral packaging mix plasmids (psPAX2 and pMD2.G) (Invitrogen) were cotransfected into HEK293T cells for 48 h in a 4:3:1 proportion. The cell cultures were harvested and used to infect fresh DF-1 cells. At 36 h after infection, the cells were passaged and selected using 10 μg/ml puromycin. The expression of VP3 protein in the DF-1 cell line was detected by immunoblotting.

### Construction of *TRAF3*-deficient HEK293T cells.

TRAF3 gene target sequences (T3-KD number 1, 5′-AGCCCGAAGCAGACCGAGTG-3′; T3-KD number 2, 5′-CCAGTTTTTGTCCCTGAACA-3′) were separately inserted into the guide RNA expression plasmid PX459 (Addgene). The recombinant constructs were separately transfected into HEK293T cells for 36 h. Cells were passaged and selected with 10 μg/ml puromycin for another 36 h. Subsequently, cells were shaken and washed with fresh DMEM and cultured with fresh DMEM containing 10% FBS for another 24 h. Finally, cells were digested and centrifuged three times and reinoculated into a new cell culture dish. The expression of TRAF3 protein was detected by immunoblotting.

### Virus titer detection.

For IBDV, fresh DF-1 cells were infected with IBDV at a multiplicity of infection (MOI) of 0.01. At different time points, cells were harvested and freeze-thawed three times. After centrifugation at 12,000 × *g* for 10 min, the supernatants were subjected to virus titer detection as described previously (57). For recombinant eGFP-VSV, cell cultures containing VSV-GFP particles were collected and serially diluted 10-fold using DMEM supplemented with 2% FBS. Then the diluted samples were used to infect fresh HEK293T cells for 48 h. The fluorescence signal of GFP protein was recorded as positive under a fluorescence microscope and calculated as the 50% tissue culture infective dose (TCID_50_) per 0.1 ml.

### Statistical analysis.

Statistical differences were assessed by one-way analyses of variance (ANOVAs) using IBM SPSS Statistics 20. The results of all statistical analyses are shown as means ± standard deviations (SD) from three independent experiments. For all experiments, a *P *of <0.05 was considered statistically significant (*, *P < *0.05; **, *P < *0.01; ***, *P < *0.001; not significant, *P > *0.05).
